# Neighborhood Environment, DNA Methylation, and Presence of Crown-Like Structures of the Breast

**DOI:** 10.1001/jamanetworkopen.2024.61334

**Published:** 2025-02-24

**Authors:** Alexandra R. Harris, Jeri D. Hughes, Wayne R. Lawrence, Petra Lenz, Jamirra Franklin, Praphulla M. S. Bhawsar, Tiffany H. Dorsey, Emily L. Rossi, Catherine M. Pichardo, Margaret S. Pichardo, Alexandra J. White, Cody Ramin, Máire A. Duggan, Mustapha Abubakar, Aaron M. Rozeboom, Jonas S. Almeida, Gretchen L. Gierach, Stefan Ambs, Brittany D. Jenkins

**Affiliations:** 1 Laboratory of Human Carcinogenesis, Center for Cancer Research, National Cancer Institute (NCI), National Institutes of Health (NIH), Bethesda, Maryland; 2 Division of Cancer Prevention, NCI, NIH, Rockville, Maryland; 3 Integrative Tumor Epidemiology Branch, Division of Cancer Epidemiology and Genetics, NCI, NIH, Rockville, Maryland; 4 Department of Biochemistry and Molecular Biology, Johns Hopkins Bloomberg School of Public Health, Baltimore, Maryland; 5 Metabolic Epidemiology Branch, Division of Cancer Epidemiology and Genetics, NCI, NIH, Rockville, Maryland; 6 Molecular Digital Pathology Laboratory, Division of Cancer Epidemiology and Genetics, NCI, Leidos Biomedical Research Inc, Frederick, Maryland; 7 Trans-Divisional Research Program, Division of Cancer Epidemiology and Genetics, NCI, NIH, Rockville, Maryland; 8 Department of Surgery, Hospital of the University of Pennsylvania, Penn Medicine, Philadelphia; 9 Epidemiology Branch, National Institute of Environmental Health Sciences, Research Triangle Park, North Carolina; 10 Department of Biomedical Sciences, Samuel Oschin Comprehensive Cancer Institute, Cedars-Sinai Medical Center, Los Angeles, California; 11 Department of Pathology and Laboratory Medicine, Cumming School of Medicine, University of Calgary, Calgary, Alberta, Canada

## Abstract

**Importance:**

Inflammation impacts cancer risk and tumor biological processes, yet studies linking it to social and environmental risk factors are lacking.

**Objective:**

To investigate the association of neighborhood deprivation and air pollution with breast adipose inflammation as well as the association between crown-like structures of the breast (CLS-B) and DNA methylation in Black and White women.

**Design, Setting, and Participants:**

This cross-sectional study analyzed women with and without breast cancer participating in the National Cancer Institute–Maryland Breast Cancer Study, most of whom were recruited between January 1, 1993, and December 1, 2003, from the University of Maryland Medical Center and surrounding hospitals in the Baltimore, Maryland, area. A small subset of the sample was recruited between March 27, 2012, and November 27, 2017. Noncancerous breast tissue was collected from women who underwent reduction mammoplasty or breast cancer surgery. Statistical analyses were conducted between May and August 2024.

**Exposures:**

Two socioenvironmental exposures were examined: air pollution (specifically, fine particulate matter less than 2.5 μm in diameter [PM_2.5_]) and neighborhood deprivation (measured with Neighborhood Deprivation Index [NDI]). Participant geocodes were linked to 2000 US Census data to calculate PM_2.5_ concentrations (total mass [μg/m^3^]) and NDI.

**Main Outcomes and Measures:**

Breast tissues underwent immunohistochemical staining for pan-macrophage marker CD68 to detect 2 outcomes: CLS-B and adipose-associated macrophages. CLS-B and adipose-related macrophages were assessed by pathologists using artificial intelligence–assisted and manual approaches. Covariate-adjusted logistic regression models were used to ascertain associations between PM_2.5_ and NDI (exposures) and presence or absence of CLS-B (outcome); CD68-positive adipose macrophages were modeled as a dichotomous high or low variable. Covariate-adjusted linear regression was used to identify associations between CLS-B (exposure) and DNA methylation (outcome).

**Results:**

The cohort included 205 participants (127 Black [62.0%], 78 White [38.0%] women; mean [SD] age, 48.7 [13.3] years). Women with vs without CLS-B had higher median (IQR) body mass index (calculated as weight in kilograms divided by height in meters squared; 35.5 [30.5-40.9] vs 31.8 [26.6-36.4]; *P* = .02). Higher levels of PM_2.5_ (odds ratio [OR], 2.32; 95% CI, 1.12-4.78; *P* = .02) and NDI (OR, 1.21; 95% CI, 1.02-1.43; *P* = .03) were associated with presence of CLS-B overall; findings were still significant among Black women (PM_2.5_: OR, 2.64 [95% CI, 1.10-6.33], *P* = .03; NDI: OR, 1.22 [95% CI, 1.01-1.48], *P* = .04) but were not statistically significant among White women (PM_2.5_: OR, 1.65 [95% CI, 0.45-5.99], *P* = .45; NDI: OR, 1.19 [95% CI, 0.83-1.70], *P* = .35). Higher PM_2.5_ concentration was associated with increased macrophage infiltration (OR, 2.11; 95% CI, 1.24-3.60; *P* = .006), with similar outcomes by race. The top 2 significant differentially methylated CpG sites by CLS-B status were *SAR1B* (β = 0.01; 95% CI, 0.01-0.02; *P* < .001) and *IL2RB* (β = −0.04; 95% CI, −0.05 to −0.02; *P* < .001). Significant interaction was observed between CLS-B status and race for *IL2RB* methylation levels (β = −0.03; 95% CI, −0.04 to −0.01; *P* for interaction <.001).

**Conclusions and Relevance:**

This cross-sectional study uncovered an association between neighborhood-level social and environmental risk factors and breast tissue inflammation. The findings help inform efforts to reduce racial and socioeconomic disparities in breast cancer and improve health equity for socially vulnerable populations.

## Key Points

**Question:**

Are neighborhood-level social and environmental risk factors associated with breast tissue macrophage infiltration, activation, and immune-related biological processes in Black and White women?

**Findings:**

In this cross-sectional study of 205 women with and without breast cancer, air pollution and neighborhood socioeconomic deprivation were associated with breast adipose macrophages and crown-like structures of the breast (CLS-B), a rare inflammatory histopathologic tissue feature associated with breast cancer risk; CLS-B were also associated with downstream epigenetic changes. These outcomes differed by race and were more robust in Black women.

**Meaning:**

The findings help inform efforts to reduce racial and socioeconomic disparities in breast cancer and to improve health equity for socially vulnerable populations.

## Teaser

This cross-sectional study examines the association of social and environmental risk factors in adipose tissue inflammation with the molecular mechanisms of developing crown-like structures of the breast among Black and White women with and without breast cancer.

## Introduction

Historically, most studies on breast cancer risk have focused on individual-level factors, such as health behaviors, socioeconomic status, and genetics.^1^ However, emerging studies indicate that neighborhood attributes may be associated with the distribution of cancer risk factors.^2,3,4^ Epidemiologic research on the neighborhood environment has increased exponentially, as neighborhoods include both physical and socioeconomic attributes that affect health. In the US, neighborhoods are strongly patterned by racial group and social position and therefore could play a role in exacerbating racial disparities in breast cancer mortality.^5,6,7^ Neighborhood deprivation, a multicomponent measure reflecting the relative socioeconomic position of neighborhoods, has been associated with a variety of social, environmental, and downstream epigenetic changes that may promote breast carcinogenesis.^3,6,8,9,10^

A common biological process that may mediate the relationship between neighborhood deprivation and risk of developing breast cancer is inflammation. Studies have uncovered associations between inflammatory factors and air quality,^11,12,13^ specifically fine particulate matter less than 2.5 μm in diameter (PM_2.5_).^14,15^ PM_2.5_ levels are a commonly used metric for air quality and are elevated in polluted areas. Large-scale reviews have observed unequal distribution of air pollution in the US, with greater exposure in poorer communities, thus implicating air pollution as a potential factor in health disparities.^16,17^ Other epidemiologic work has linked PM_2.5_ with breast cancer incidence and aggressiveness.^14,18,19,20^ However, more exploration is needed into how inflammation may play a role in mediating changes to breast tissue and promoting breast cancer, including investigation at the molecular level.

Composed of dead or dying adipocytes surrounded by CD68-positive macrophages, crown-like structures of the breast (CLS-B) are considered as a hallmark of adipose tissue inflammation and have been shown to be associated with obesity, older age, unfavorable metabolic profile, and increased breast cancer risk.^21,22,23,24,25^ However, the occurrence of CLS-B has not been studied in the context of socioenvironmental factors. Investigating CLS-B in the context of the neighborhood environment could increase knowledge of the heightened breast cancer risk among women from socioeconomically vulnerable communities. Additionally, PM_2.5_ and neighborhood deprivation have both been associated with epigenetic modifications that may impact breast tumorigenesis,^9,26,27^ but the relationship between DNA methylation and CLS-B remains unexplored. Here, we aimed to investigate the association of neighborhood deprivation and air pollution with breast adipose inflammation as well as the association between CLS-B and DNA methylation in Black and White women.

## Methods

### Study Population and Data Collection

This cross-sectional study included a convenience sample of women with and without breast cancer participating in the National Cancer Institute–Maryland Breast Cancer Study who donated noncancerous tissue from breast surgeries. All participants provided written informed consent. The study as well as the biospecimen and data collection were approved by the University of Maryland Institutional Review Board for the participating institutions (University of Maryland Medical Center and 4 surrounding hospitals in the Baltimore, Maryland, area) and by the National Institutes of Health Office for Human Research Protections. We followed the Strengthening the Reporting of Observational Studies in Epidemiology (STROBE) reporting guideline.

After exclusions (eMethods in Supplement 1), the final analytic population included participants who donated reduction mammoplasty or normal-adjacent tissue. All participants completed interviewer-administered questionnaires on breast cancer risk factors of interest, including demographic characteristics. Due to the socioeconomic relevance of this work, we herein described race as a social construct (ie, Black or White), which was self-reported by participants answering the questionnaire. Recruitment focused on Black and White women due to the large representation of these 2 population groups in the Baltimore, Maryland, area. Additionally, due to the high breast cancer mortality rate among Black women in the US, we examined the biological impact of socioenvironmental factors both regardless of race and stratified by race. Most participants (82%) were recruited between January 1, 1993, and December 1, 2003, with a small subset of samples (18%) recruited between March 27, 2012, and November 27, 2017.

### Air Pollution and Neighborhood Deprivation Index

PM_2.5_ concentrations (total mass [μg/m^3^]) were ascertained at a 12-by-12-km grid level and linked to each participant’s geocoded residential address at the time of study enrollment as previously described.^28^ PM_2.5_ concentration for 2000 was measured using the Environmental Protection Agency downscaling model^29^ (eMethods in Supplement 1).

Construction of the Neighborhood Deprivation Index (NDI) has been described previously.^30^ In brief, Census tract–level socioeconomic deprivation was measured from the 2000 US Census using the NDI developed by Messer et al.^31^ Domains used to create the NDI were measured at the Census-tract level, which is widely considered to be the best approximation of a neighborhood.^32,33^ Using principal component analysis, we extracted a single factor representing the shared variance from the deprivation indicators in the 2000 Census. Six variables with a principal component analysis loading value above 0.25 were retained in the NDI: percentage of households living in poverty, percentage of households receiving public assistance, percentage of female-headed households with dependent children, percentage of households earning less than $30 000 per year, percentage of males and females unemployed, and percentage of households with no car (eFigure 1 in Supplement 1). Lower deprivation was indicated by lower NDI values, whereas higher deprivation was indicated by higher NDI values. Additional details can be found in the eMethods in Supplement 1.

### Immunohistochemistry

Sections of formalin-fixed, paraffin-embedded blocks from adjacent noncancerous and mammoplastic reduction tissue specimens were stained with anti-CD68 antibody, digitized, and imported into the HALO software (Indica Labs) to undergo image analysis. The eMethods in Supplement 1 provides more information.

### Quantification of CLS-B 

**Figure 1.**
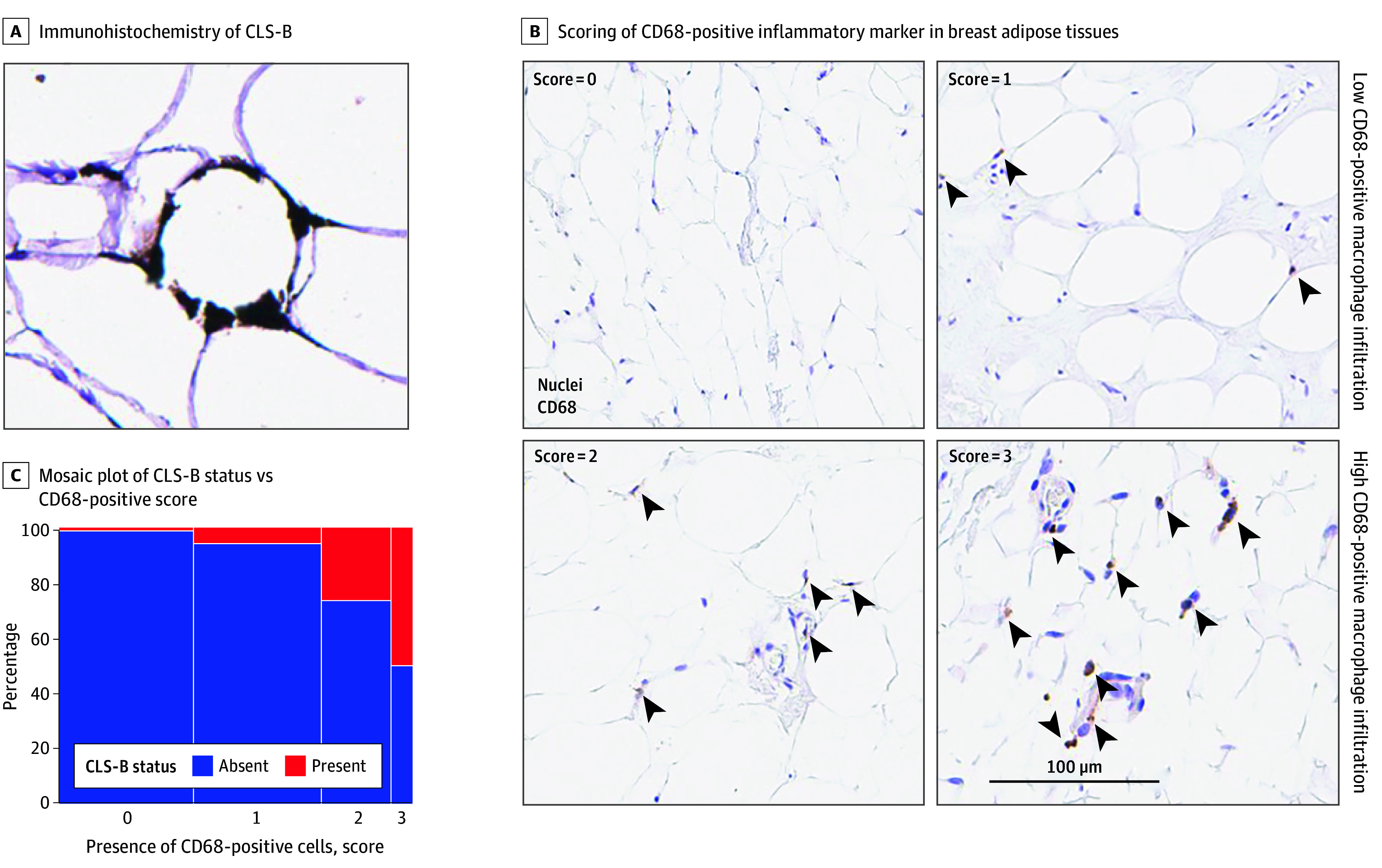
Representative Images of and Associations Between Crown-Like Structures of the Breast (CLS-B) and CD68-Positive Macrophage Infiltrates A, A complete CLS-B is illustrated by CD68-positive macrophages (brown) encircling an adipocyte. B, Increasing levels of CD68-positive macrophage infiltration (score of 0 indicates rare, 1 indicates low, 2 indicates moderate, and 3 indicates dense). Scores 0 and 1 were then classified as low levels of CD68-positive adipose macrophages, while scores 2 and 3 were classified as high levels. Arrowheads indicate CD68-positive macrophages. C, Comparison of CLS-B status with CD68-positive score had a *P* < .001, which was ascertained using a χ^2^ test.

CLS-B are identified morphologically by the presence of a ring of CD68-positive mononuclear cells surrounding an adipocyte (Figure 1A). CLS-B are rare, and microscope-based detection by a pathologist is time consuming. In this study, pathologists quantified CLS-B in breast tissues through an artificial intelligence (AI)–assisted approach using a recently developed convolutional neural network trained to identify CLS-B from digitized whole slide images.^34^ Presence of CLS-B (borderline and complete) was defined as having 1 or more CLS-B detected in adipose breast tissue. CLS-B status was modeled as a dichotomous variable (present vs absent). Additional details are available in the eMethods in Supplement 1.

### Quantification of CD68-Positive Macrophage Infiltrates in Adipose Tissues

To quantify CD68-positive macrophages in breast adipose tissue, scanned images were reviewed in the HALO link and manually scored for the presence of CD68-positive cells on a scale of 0 to 3 (0 = rare, 1 = low, 2 = moderate, 3 = dense) by a trained pathologist (P.L.) (Figure 1B). Presence of CLS-B was associated (*P* < .001) with higher levels of CD68-positive adipose macrophages (Figure 1C). Scores 0 and 1 were classified as a low level of CD68-positive adipose macrophages, and scores 2 and 3 were classified as a high level of CD68-positive adipose macrophages. Henceforth, CD68-positive adipose macrophages were modeled as a dichotomous variable (high vs low). The eMethods in Supplement 1 provides more detail.

### DNA Methylation and Functional Enrichment Analysis

DNA methylation levels were estimated using the Infinium MethylationEPIC 850K BeadChip V.1.0 (Illumina Inc) according to the manufacturer’s protocol. Methylation preprocessing, quality control metrics, and normalization were previously described.^9^ The approach we used yielded a total of 750 426 probes for the final methylation analysis. After sorting based on false discovery rate, we identified the 2 most significant differentially methylated CpG sites associated with CLS-B (cg21799270 within the *SAR1B* [secretion-associated Ras-related GTPase 1B] gene promoter and cg10040153 within the *IL2RB* [interleukin 2 (IL-2) receptor subunit β] gene promoter) to explore for further downstream analyses.

The top 2 differentially methylated genes (*SAR1B* and *IL2RB*) were subjected to gene ontology (GO) pathway annotation. The analysis of biological process GO terms was performed with the Enrichr interface,^35^ a widely used enrichment analysis tool that summarizes collective functions of gene lists and individual genes using gene set libraries. The top 18 GO terms were sorted according to their log-transformed *P* values.

### Statistical Analysis

Multivariable logistic regression modeling was used to analyze whether the presence of CLS-B (dichotomous) differed by 2 primary exposures of interest: NDI (continuous) and PM_2.5_ levels (continuous). Because PM_2.5_ levels were not normally distributed, a zero-skewness log transformation was performed prior to subsequent regression analyses. The same modeling approach was used for CD68-positive adipose macrophages (dichotomous). Multivariable linear regression modeling was used to test associations between individual CpG sites of interest (outcome) and CLS-B status (exposure), with additional testing for multiplicative statistical interaction between CLS-B and race for cg10040153.

All regression models were adjusted for participant age (continuous), race (categorical), and body mass index (BMI [calculated as weight in kilograms divided by height in meters squared]; continuous), where appropriate; these variables were selected a priori based on known associations with CLS-B.^24,25,36,37,38,39^ CLS-B status did not vary statistically significantly by tissue type (normal-adjacent vs reduction mammoplasty), year of surgery, educational level, diabetes, menopausal status, smoking, alcohol consumption, or study recruitment phase (1993-2003 vs 2012-2019); thus, these variables were not included as covariates in regression models to improve model parsimony. Subsequent stratification by participant race (White [referent] vs Black) was performed for each exposure.

Results are presented as adjusted estimates with corresponding 95% CIs. All statistical tests were 2-sided, with statistical significance set at *P* < .05. Statistical analyses were conducted between May and August 2024 using Stata, version 18.0 (StataCorp LLC) except methylation analyses, which were conducted using JMP, version 18.0.1 (JMP Statistical Discovery LLC).

## Results

### Participant Characteristics

Of the 205 participants, 127 (62.0%) self-identified as Black and 78 (38.0%) as White women with a mean (SD) age of 48.7 (13.3) years. Characteristics of participants in the analytic cohort are shown by the presence (21 [10.2%]) or absence (184 [89.8%]) of CLS-B in Table 1. Women with CLS-B were more likely to self-identify as Black than White (14 [66.7%] vs 7 [33.3%]), although the difference was not statistically significant. Women with vs without CLS-B had higher median (IQR) BMI (35.5 [30.5-40.9] vs 31.8 [26.6-36.4]; *P* = .02). The mean (range) log-transformed PM_2.5_ concentration for the study population was −2.64 (−4.07 to −1.12) μg/m^3^; the mean (range) NDI was 1.44 (−2.60 to 9.47) (eFigure 2A and C in Supplement 1). While the mean (range) PM_2.5_ concentrations did not vary significantly by race (Black: −2.63 [−4.07 to −1.12] μg/m^3^; White: −2.64 [−3.94 to −1.25] μg/m^3^; *P* = .91), the mean (range) NDI was higher in Black women than White women (2.75 [−2.27 to 9.47] vs −0.68 [−2.60 to 6.64]; *P* < .001) (eFigure 2B and D in Supplement 1).

**Table 1.** Participant Characteristics Stratified by Crown-Like Structures of the Breast Status

Characteristics	Participants, No. (%)		
	Total (N = 205)	CLS-B absent (n = 184)	CLS-B present (n = 21)
Demographics			
Age, mean (SD), y	48.7 (13.3)	48.6 (13.0)	49.6 (15.4)
BMI, median (IQR)	31.8 (27.2-36.9)	31.8 (26.6-36.4)	35.5 (30.5-40.9)
Self-reported race^a^			
Black	127 (62.0)	113 (61.4)	14 (66.7)
White	78 (38.0)	71 (38.6)	7 (33.3)
Educational level			
<High school	22 (10.7)	18 (9.8)	4 (19.0)
High school diploma	54 (26.3)	50 (27.2)	4 (19.0)
College degree	13 (6.3)	13 (7.1)	0
Graduate school	13 (6.3)	12 (6.5)	1 (4.8)
Unknown	103 (50.2)	91 (49.5)	12 (57.1)
Health factors			
Ever smoker^b^			
Yes	74 (36.1)	65 (35.3)	9 (42.9)
No	83 (40.5)	74 (40.2)	9 (42.9)
Unknown	48 (23.4)	45 (24.5)	3 (14.3)
Alcohol use			
Yes	86 (42.0)	73 (40.0)	13 (61.9)
No	69 (33.7)	64 (34.8)	5 (23.8)
Unknown	50 (24.4)	47 (25.5)	3 (14.3)
Diabetes			
Yes	25 (12.2)	20 (10.9)	5 (23.8)
No	130 (63.4)	117 (63.6)	13 (61.9)
Unknown	50 (24.4)	47 (25.5)	3 (14.3)
Family history of breast cancer			
Yes	18 (8.8)	16 (8.7)	2 (9.5)
No	135 (65.9)	120 (65.2)	15 (71.4)
Unknown	52 (25.4)	48 (26.1)	4 (19.0)
Menopause			
Yes	56 (27.3)	48 (26.1)	8 (38.1)
No	74 (36.1)	66 (35.9)	8 (38.1)
Unknown	75 (36.6)	91 (49.5)	5 (23.8)
Breast tissue type			
Reduction mammoplasty	101 (49.3)	90 (48.9)	11 (52.4)
Normal-adjacent	104 (50.7)	94 (51.1)	10 (47.6)
Unknown	0	0	0

Abbreviations: BMI, body mass index (calculated as weight in kilograms divided by height in meters squared); CLS-B, crown-like structures of the breast.

^a^ Recruitment focused on Black and White women due to the large representation of these 2 population groups in the Baltimore, Maryland, area. Additionally, due to the high breast cancer mortality rate among Black women in the US, we examined the biological impact of socioenvironmental factors both regardless of race and stratified by race.

^b^ Smoking status describes cigarette smoking.

### Air Pollution and CLS-B

We first examined the association between air pollution (specifically PM_2.5_) and CLS-B in all participants, adjusting for age, BMI, and race. Higher PM_2.5_ levels were associated with an increase in the odds of detecting CLS-B (odds ratio [OR], 2.32; 95% CI, 1.12-4.78; *P* = .02) (Table 2). When we further stratified by race, we detected an association between PM_2.5_ and CLS-B in Black women (OR, 2.64; 95% CI, 1.10-6.33; *P* = .03) but not in White women (OR, 1.65; 95% CI, 0.45-5.99; *P* = .45), although the directionality of the association was the same regardless of race.

**Table 2.** Association Between Socioenvironmental Exposures and Breast Adipose–Associated Macrophages

	All participants^a^		White		Black	
	OR (95% CI)	*P* value	OR (95% CI)	*P* value	OR (95% CI)	*P* value
**Air pollution and CLS-B** ^b^						
No.	200	NA	73	NA	127	
PM_2.5_, μg/m^3^^c^	2.32 (1.12-4.78)	.02	1.65 (0.45-5.99)	.45	2.64 (1.10-6.33)	.03
**Air pollution and CD68-positive macrophages in adipose tissue** ^d^						
No.	190	NA	70	NA	120	
PM_2.5_, μg/m^3^^c^	2.11 (1.24-3.60)	.006	3.76 (1.30-10.87)	.02	1.72 (0.91-3.25)	.09
**Neighborhood deprivation and CLS-B** ^b^						
No.	205	NA	78	NA	127	
NDI^e^	1.21 (1.02-1.43)	.03	1.19 (0.83-1.70)	.35	1.22 (1.01-1.48)	.04
**Neighborhood deprivation and CD68-positive macrophages in adipose tissue** ^d^						
No.	194	NA	74	NA	120	
NDI^e^	1.02 (0.90-1.15)	.79	1.04 (0.77-1.41)	.78	1.01 (0.88-1.16)	.88

Abbreviations: CLS-B, crown-like structures of the breast; NDI, Neighborhood Deprivation Index; OR, odds ratio; PM_2.5_, fine particulate matter.

^a^ Unstratified models adjusted for age, body mass index (BMI), and race. Stratified models adjusted for age and BMI.

^b^ CLS-B status: present vs absent.

^c^ PM_2.5_: log-transformed, continuous.

^d^ CD68-positive macrophages in adipose tissue: high vs low.

^e^ NDI: continuous.

Given the known role of macrophages in breast carcinogenesis and progression, we investigated whether air pollution was associated with infiltration of CD68-positive macrophages in breast adipose tissue. Across all participants, we observed significantly higher CD68-positive adipose macrophages with higher PM_2.5_ levels (OR, 2.11; 95% CI, 1.24-3.60; *P* = .006). Further stratification by race revealed an association between PM_2.5_ levels and adipose macrophages in White women (OR, 3.76; 95% CI, 1.30-10.87; *P* = .02) but not in Black women (OR, 1.72; 95% CI, 0.91-3.25; *P* = .09) (Table 2). Altogether, these data suggest that higher exposure to air pollutants may induce macrophage-related inflammatory changes within the breast immune microenvironment, which may differ by race.

### Neighborhood Deprivation and CLS-B

We used a similar approach to identify the association of NDI with the development of CLS-B. Overall, higher NDI was associated with an increase in the odds of detecting CLS-B (OR, 1.21; 95% CI, 1.02-1.43; *P* = .03) (Table 3). When further stratified by race, the association between NDI and CLS-B was detected only in Black women (OR, 1.22; 95% CI, 1.01-1.48; *P* = .04) but not in White women (OR, 1.19; 95% CI, 0.83-1.70; *P* = .35); however, the directionality of association was the same regardless of race and the difference in significance may be impacted by statistical power within subgroups. We did not observe an association between NDI and total CD68-positive adipose macrophages in the overall and race-stratified analyses. The data pointed to a moderate magnitude of association between living in a neighborhood with higher NDI (deprivation) and developing CLS-B. Furthermore, these outcomes were most pronounced in Black women.

**Table 3.** Association Between CLS-B Status and Methylation of CpG Sites

CpG site	Gene	β (95% CI)^a^	*P* value	*P* value for interaction
**CLS-B status and methylation of CpG sites^b^**				
cg21799270	*SAR1B*	0.01 (0.01 to 0.02)	<.001	NA
cg10040153	*IL2RB*	−0.04 (−0.05 to −0.02)	<.001	NA
**Race and methylation of CpG sites^b^**				
cg21799270	*SAR1B*	0.003 (0.0003 to 0.006)	.03	NA
cg10040153	*IL2RB*	−0.01 (−0.02 to −0.005)	.002	NA
**CLS-B status with race interaction and methylation of CpG sites^a^**				
cg21799270	*SAR1B*	−0.003 (−0.007 to 0.001)	NA	.17
cg10040153	*IL2RB*	−0.03 (−0.04 to −0.01)	NA	<.001

Abbreviations: CLS-B, crown-like structures of the breast; NA, not applicable.

^a^ Additional adjustment for interaction term between CLS-B status and race.

^b^ Linear regression model included CLS-B status (present vs absent), race (Black vs White), age (continuous), body mass index (continuous), and methylation batch (categorical).

### DNA Methylation and CLS-B

To explore the molecular processes involved in CLS-B development in breast tissue, we investigated differentially methylated CpG sites in relation to the presence of CLS-B. The top 2 differentially methylated CpG sites by CLS-B status were in the promoter regions of *SAR1B* (cg21799270) and *IL2RB* (cg10040153).

**Figure 2.**
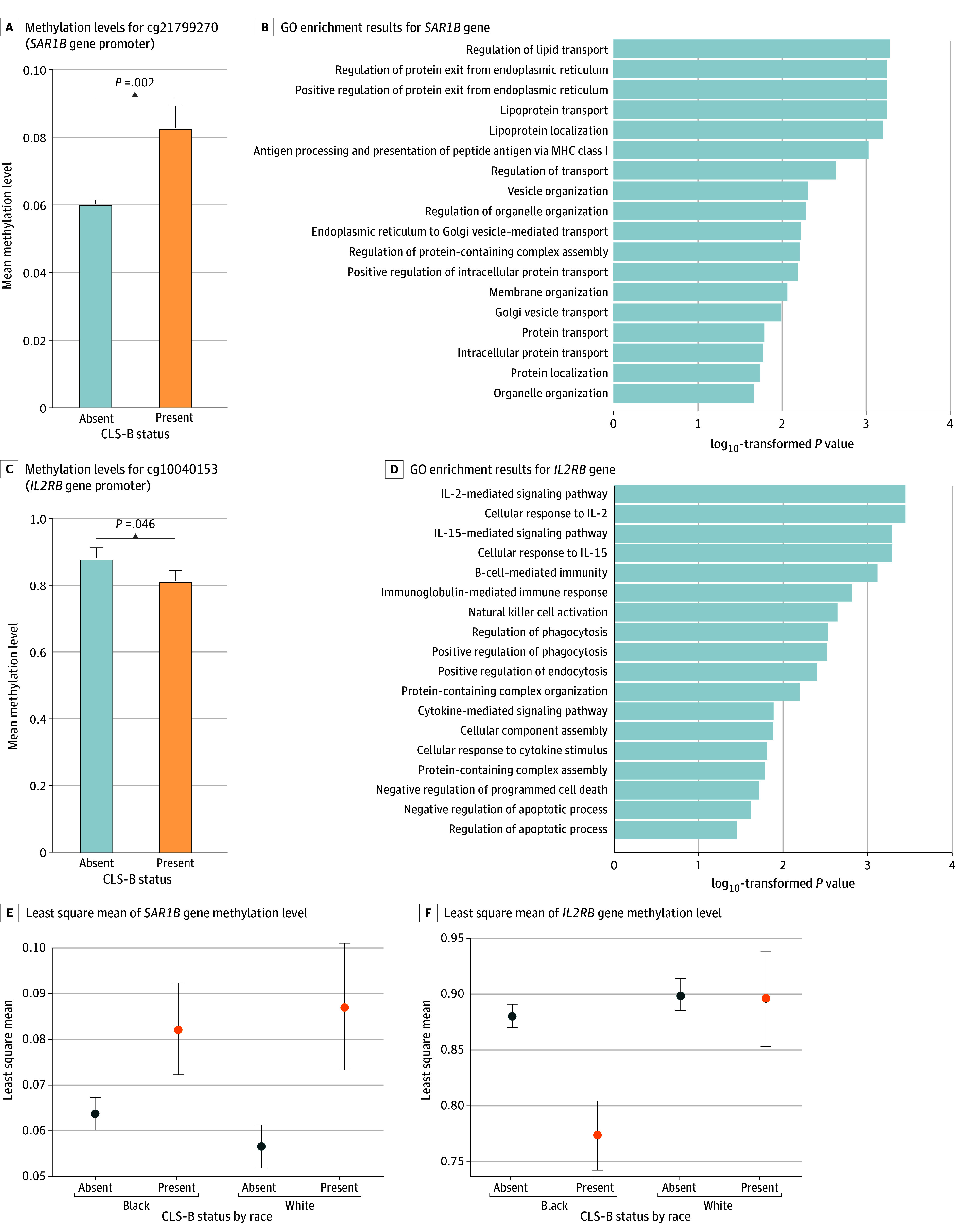
Methylation of *SAR1B* and *IL2RB* Genes in Women With Crown-Like Structures of the Breast (CLS-B) Error bars represent SEs. GO indicates gene ontology; IL-2, interleukin 2; MHC, major histocompatibility complex.

Methylation levels of *SAR1B*, an intracellular leucine sensor and regulator of mammalian target of rapamycin complex 1 (mTORC1) signaling, were significantly higher in women with CLS-B compared with women without CLS-B (mean difference, 0.02; *P* = .002) (Figure 2A). To better understand the underlying biological processes associated with the expression of these genes, we performed functional enrichment analyses. *SAR1B* showed significant enrichment of 18 GO processes, which were largely related to lipid transport and antigen presentation via major histocompatibility complex class I (Figure 2B). Conversely, methylation levels of *IL2RB* were significantly lower in women with CLS-B compared with women without CLS-B (mean difference, −0.07; *P* = .046) (Figure 2C). Functional enrichment analysis of *IL2RB* revealed its involvement in 18 GO processes, which included numerous cytokine signaling pathways and immune response processes (eg, IL-2 and IL-15 signaling and cellular responses; B-cell– and NK-cell–mediated immunity). Particularly relevant to CLS-B, *IL2RB* was linked to negative regulation of apoptosis and increased phagocytosis by immune cells (Figure 2D).

In multivariable linear regression models adjusted for age, BMI, and race, cg21799270 and cg10040153 CpG sites showed associations with CLS-B status (*SAR1B*: β = 0.01 [95% CI, 0.01-0.02], *P* < .001; *IL2RB*: β = −0.04 [95% CI, −0.05 to −0.02], *P* < .001) (Figure 2E, Table 3). Sensitivity analyses that additionally adjusted for PM_2.5_ concentration and NDI did not show attenuation of the association between methylation of these CpG sites and CLS-B status. Furthermore, both CpG sites of interest were associated with race, with *SAR1B* methylation levels higher in Black women (β = 0.003; 95% CI, 0.0003-0.006; *P* = .03) and *IL2RB* methylation levels lower in Black women (β = −0.01; 95% CI, −0.02 to −0.005; *P* = .002) (Table 3). We tested whether there was a significant interaction between CLS-B status and race in the methylation levels of either CpG site. While we did not observe a significant interaction between CLS-B status and race with *SAR1B* methylation levels (β for interaction = −0.003; 95% CI, −0.007 to 0.001; *P* for interaction = .17), we found a significant interaction for *IL2RB* methylation levels (β for interaction = −0.03; 95% CI, −0.04 to −0.01; *P* for interaction <.001) (Figure 2E and F, Table 3). In women without CLS-B, methylation levels of *IL2RB* were comparably high by race. However, in women with CLS-B, Black women showed lower methylation levels of *IL2RB,* while White women showed significantly higher methylation levels; these data suggest that the biological underpinnings of CLS-B may differ by race.

## Discussion

In this study, we explored the association between socioenvironmental risk factors and macrophage-mediated adipose tissue inflammation in a diverse cohort of women from the Baltimore area. We found that higher PM_2.5_ concentrations were associated with increased odds of detecting CLS-B and higher levels of macrophage infiltration into breast adipose tissue. Similarly, residing in a neighborhood with higher deprivation was also associated with increased odds of detecting CLS-B. Furthermore, we uncovered associations between the presence of CLS-B and cancer- and immune-related gene methylation, including interactions with race. To our knowledge, this study was the first to link neighborhood environment, DNA methylation, and macrophage-mediated tissue inflammation within the breast. This work provides novel insights into how neighborhood deprivation and air pollution play a role in modulating breast tissue inflammation.

This study adds to the increasing body of evidence that air pollution can physiologically affect breast tissue composition. To date, PM_2.5_ has been associated with decreased involution of terminal ductal lobular units, a tissue feature associated with breast cancer risk.^28^ The present findings build on earlier evidence, showing that air pollutants can deleteriously impact not only the parenchymal epithelia but also adipocytes and immune cells within the breast. Thus, the association of chronic exposure to fine particulates and neighborhood socioeconomic environment with breast pathophysiological processes and related tissue inflammation is a plausible link between these types of histologic features and breast cancer risk.^26,27^ These findings indicate that the biological implications of neighborhood-level exposures for adipose tissue macrophages may differ by race. The association between CLS-B and self-reported race has been explored previously,^25,36^ although these limited studies used different markers and did not link CLS-B with socioenvironmental factors. Conflicting reports on CLS-B are often complicated by differences in study design and methods of CLS-B measurement and evaluation,^40^ supporting the need for a standardized and validated algorithm to study CLS-B more broadly.^34^

Multiple studies have evaluated the association between obesity (BMI ≥30) and the presence of CLS-B.^24,41,42,43,44^ We also observed an association between BMI and CLS-B in this study’s cohort, confirming this important relationship. The presence of CLS-B was associated with higher levels of CD68-positive adipose macrophages (Figure 1C). While intuitive, no CLS-B were detected in half of the participants with the highest levels of CD68-positive adipose macrophages (ie, score of 3). This finding suggests that, even when macrophages have infiltrated the breast adipose tissue, additional activating factors are needed to instigate their formation into CLS-B.

In examining the molecular processes underlying CLS-B development in breast tissue, we assessed DNA methylation. Methylation levels of the promoter region of *SAR1B* were increased in women with CLS-B, which would generally be associated with a decrease in gene expression. *SAR1B* is known to be involved in intracellular leucine sensing, lipid transport, and negative regulation of mTORC1 signaling, a major intracellular pathway central to cancer initiation and progression.^45,46^ Particularly relevant to these findings is that mTORC1 signaling is activated in high adiposity tissues and actively participates in lipid deposition and adipose tissue expansion.^46,47^ Thus, it is plausible that a potential decrease in *SAR1B* expression could play a role in inducing formation of CLS-B, although more mechanistic work is needed.

*IL2RB* methylation was decreased in women with CLS-B, suggesting an increase in gene expression for these individuals. *IL2RB* is a cytokine receptor for IL-2 and is associated with the infiltration of immune cells, including high expression in a subset of CD68-positive breast macrophages.^48^ Several studies have indicated both immunosuppressive and immunostimulatory roles of IL-2 or *IL2RB*, depending on the context of the tumor microenvironment,^49,50^ but dysregulated *IL2RB* expression has been associated with impaired immune response and cancer susceptibility.^51^ More studies are needed to decipher the exact role of *IL2RB* in breast carcinogenesis, specifically as it relates to adipose tissue and CLS-B. Additionally, we observed a significant interaction between race and CLS-B in the methylation of *IL2RB*, with only Black women with CLS-B showing decreased methylation of this gene. This finding could suggest that the biological mechanisms governing CLS-B formation may differ by race, although more mechanistic investigation is warranted. However, this study marks an important first step toward characterizing the relationship between DNA methylation patterns and occurrence of CLS-B across different population groups.

### Strengths and Limitations

This study has several strengths. There was racial diversity in the cohort, which was drawn from a geographic area that bears a disproportionate burden of social and environmental risk factors for disease. We also used a more streamlined, pathologist-validated, artificial intelligence–assisted approach to detect CLS-B, addressing a major challenge in the field. Furthermore, to our knowledge, this study was the first to examine the association between CLS-B and DNA methylation, providing novel mechanistic insights into CLS-B development.

There are several study limitations. We were unable to analyze exposure to air pollution and NDI over the life course due to the cross-sectional nature of this study. Furthermore, the rarity of CLS-B made achieving adequate statistical power challenging. Additionally, we recognize that the normal-adjacent and reduction mammoplasty breast tissues analyzed in this study are not necessarily representative of true normal breast tissue. As such, additional studies into the molecular underpinnings of CLS-B development are needed to confirm and expand these findings, especially differential outcomes by race.

## Conclusions

In this cross-sectional study, we investigated the association of breast tissue histologic markers of inflammation with neighborhood-level social and environmental risk factors and DNA methylation. These findings can help inform the ongoing efforts to reduce racial and socioeconomic disparities in breast cancer and improve overall health equity for socially vulnerable populations.

## Notes

Supplement 1.
eMethods

eReferences
**eFigure 1.** Deprivation Indicators From the 2000 Census Used to Construct Neighborhood Deprivation Index **eFigure 2.** Distribution of Main Exposures in Study Population

Supplement 2.
Data Sharing Statement
