# Supplementary material for: Neighborhood Environment, DNA Methylation, and Presence of Crown-Like Structures of the Breast
Source: JAMA Netw Open. 2025 Feb 24;8(2):e2461334. doi: 10.1001/jamanetworkopen.2024.61334 (PMC11851241; doi:10.1001/jamanetworkopen.2024.61334)
Supplement: Supplement 1. — eMethods eReferences eFigure 1. Deprivation Indicators From the 2000 Census Used to Construct Neighborhood Deprivation Index eFigure 2. Distribution of Main Exposures in Study Population [file jamanetwopen-e2461334-s001.pdf]

Supplemental Online Content

Harris AR, Hughes JD, Lawrence WR, et al. Neighborhood environment, DNA methylation, and presence of crown-like structure of the breast. *JAMA Netw Open*. 2025;8(2):e2461334. doi:10.1001/jamanetworkopen.2024.61334

eMethods

eReferences

eFigure 1. Deprivation Indicators From the 2000 Census Used to Construct Neighborhood Deprivation Index

eFigure 2. Distribution of Main Exposures in Study Population

This supplemental material has been provided by the authors to give readers additional information about their work.

© 2025 Harris AR et al. *JAMA Network Open*

eMethods

Study population and Data Collection. The NCI-Maryland Breast Cancer Study is a cross-sectional, molecular epidemiologic study initiated through a long-standing contract between NCI and the University of Maryland. Of the 456 participants, we excluded samples from those who did not have available normal (reduction or normal adjacent) tissue (n=241) or did not have geocoded data for linkage to exposures (n=10), leaving 205 participants for our analyses; 101 tissues were from reduction mammoplasty (n=61 Black, n=40 White) and 104 were from normal-adjacent breast tissue samples (n=66 Black, n=38 White). The median was used to impute missing BMI for 9 participants. All study participants signed written informed consent upon study entry and completed interviewer-administered questionnaires of breast cancer risk factors of interest including demographic characteristics.

Air pollution. $PM_{2.5}$ concentration for 2000 was measured using the Environmental Protection Agency (EPA) Downscale Model[1]. The EPA's Downscale model is a Baysian space-time downscaling fusion model that merges modeled air pollutant concentration from the Community Multiscale Air Quality System with air pollutant measurements captured from air monitoring stations. 5 participants were missing air pollution data due to incomplete geocoding and were excluded in regression analyses for which air pollution was the primary exposure.

Neighborhood Deprivation Index. Neighborhood-level data were drawn from the 2000 Census using the Neighborhood Change Database[2], which adjusts for tract boundary changes between censuses.

Immunohistochemistry. Adjacent non-cancerous tissue specimens were collected from breast cancer patients at the time of cytoreductive surgery (n=104). Non-cancerous breast tissues were also obtained from women undergoing a breast reduction procedure (n=101). All samples were routinely processed by pathologists immediately following surgery at the Department of Pathology, University of Maryland (Baltimore, MD). Formalin- fixed and paraffin-embedded (FFPE) tissue blocks were sectioned at 5-micron thickness. A representative section from each tissue block was hematoxylin-eosin (H&E) stained, digitized, and used to confirm no cancerous tissue was present. Additional sections of the selected FFPE blocks were cut and stained with anti-

© 2025 Harris AR et al. *JAMA Network Open*

CD68 (KP-1) primary antibody (Code# 790-2931, Lot# K08454) with the DISC. ChromoMap DAB RUO kit (Code# 760-159) according to the manufacturer's guidelines. All sections were processed on the Ventana Discovery Ultra automated staining platform together with a positive control slide for each staining run. All slides were stained by the Molecular and Digital Pathology Lab in NCI Division of Cancer Epidemiology and Genetics (DCEG) and then digitized, and imported into HALO© software (Halo, Indica Labs, Albuquerque, New Mexico) to undergo image analysis.

Quantification of crown-like structures of the breast. The recently developed convolutional neural network was trained on an initial dataset of images with CLS-B presence confirmed by agreement amongst 3 reviewers (PL, MA, MD). Following additional modelling and active learning, the network was retrained and validated by 2 reviewers. The final model achieved an ROC AUC of 0.90. and greatly reduced the time and effort required by a pathologist to identify CLS-B. The model does not distinguish between complete and borderline CLS-B. It is available for use via the epiPath web application designed by the NCI DCEG Data Science & Engineering Research Group (https://episphere.github.io/path). Full methodology of CLS-B screening using this tool can be found in Bhawsar et al[3].

Quantification of CD68+ macrophage infiltrates in adipose tissues. Average numbers of CD68+ cells per mm^2 of adipose tissue area for each score are as follows: score 0 (rare) = 15 CD68+ cells/mm^2 adipose areas; score 1 (low) = 34 CD68+ cells/mm^2 adipose area; score 2 (moderate) = 59 CD68+ cells/mm^2 adipose area; score 3 (dense) = 97 CD68+ cells/mm^2 adipose area. Thus, a score of 0 does not mean the whole tissue is completely devoid of any macrophages; a score of 0 means that >90% of adipose areas viewed at high power did not contain CD68+ cells.

eReferences

1. Environmental Protection Agency. *Technical Information About Fused Air Quality Surface Using Downscaling Tool: Metadata Description*; 2016.
2. Neighborhood Change Database. GeoLytics; 2014. Accessed July 1, 2021.

© 2025 Harris AR et al. *JAMA Network Open*

3. Bhawsar, P MS. *et a*l. Crown-Like Structures in Breast Adipose Tissue: Finding a 'Needle-in-a- Haystack' using Artificial Intelligence and Collaborative Active Learning on the Web. arXiv:2409.08275 [q-bio.QM]. https://doi.org/10.48550/arXiv.2409.08275.

© 2025 Harris AR et al. *JAMA Network Open*

Employment variables	*Housing variables*
**Percent males and females unemployed**, Percent males no longer in workforce	Percent rented, percent vacant, percent crowded, percent renter or owner costs in excess of 50% of income, median owner household value
Occupation variables	*Poverty variables*
Percent males and females in management, percent males, females in professional occupations	**Percent households in poverty**, **percent female headed households with dependent children**, **percent households earning under $30,000/year, percent households on public assistance, percent households with no car**
Racial composition	*Residential stability variables*
Percent residents who were non-Hispanic Blacks	Percent in same residence since 1995, percent residents 65 years and above

eFigure 1. **Deprivation indicators from the 2000 Census used to construct neighborhood deprivation index (NDI, bolded).**

© 2025 Harris AR et al. *JAMA Network Open*

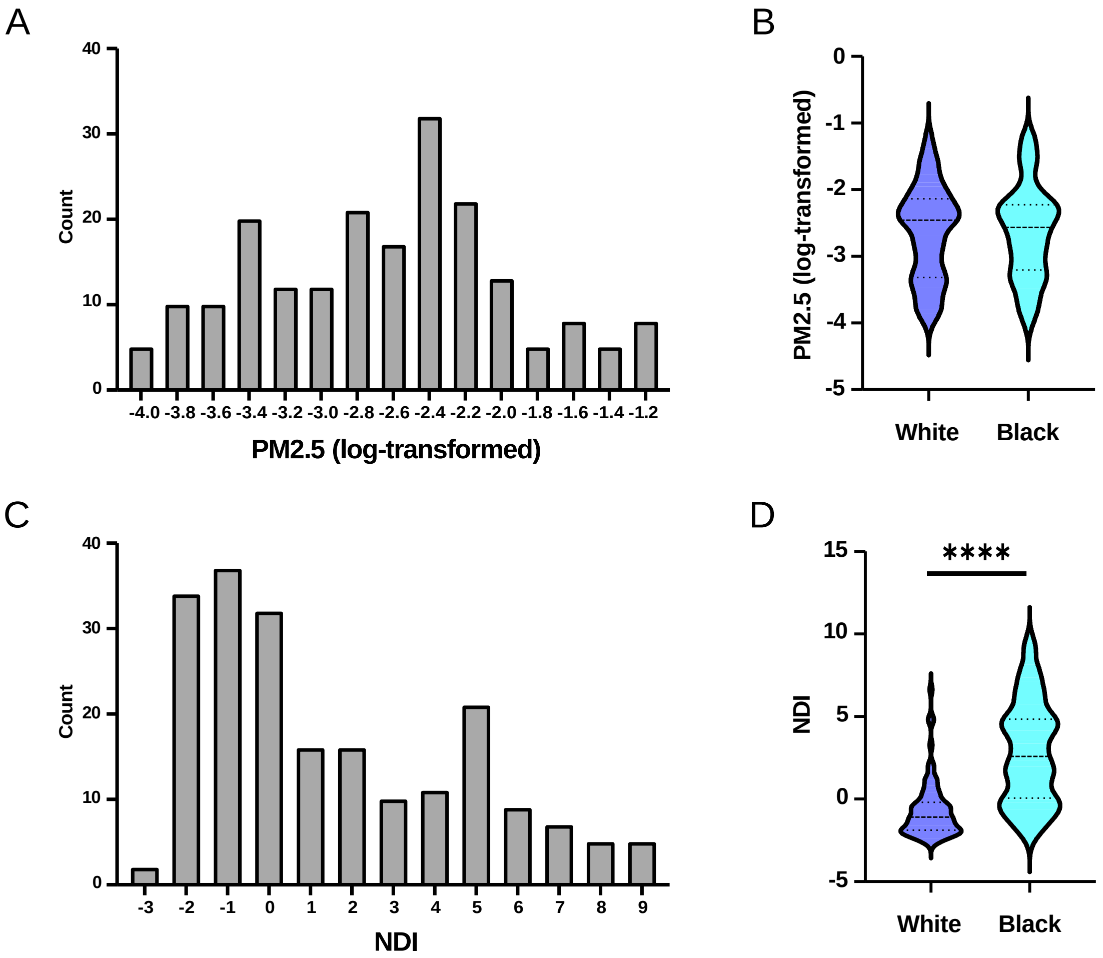

eFigure 2. Distribution of main exposures in study population. (A) The distribution of $PM_{2.5}$ concentration (log-transformed) for our study population (mean [range]: -2.64 [-4.07 to -1.12]). (**B**) The distribution of NDI for our study population (mean [range]: 1.44 [-2.60 to 9.47]). **(C)** Mean $PM_{2.5}$ concentrations by race (Black: -2.63 [-4.07 to -1.12], White: -2.64 [-3.94 to -1.25], *P*=0.91). **(D)** Mean NDI by race (Black: 2.75 [-2.27 to 9.47], White: -0.68 [-2.60 to 6.64], *P*<0.0001). Student's T-test, **** *P*<0.0001.

© 2025 Harris AR et al. *JAMA Network Open*
