# Supplementary material for: Neighborhood Environment, DNA Methylation, and Presence of Crown-Like Structures of the Breast
Source: JAMA Netw Open. 2025 Feb 24;8(2):e2461334. doi: 10.1001/jamanetworkopen.2024.61334 (PMC11851241; doi:10.1001/jamanetworkopen.2024.61334)
Supplement: Supplement 2. — Data Sharing Statement [file jamanetwopen-e2461334-s002.pdf]

Data Sharing Statement

Harris. Neighborhood Environment, DNA Methylation, and Presence of Crown-Like Structures of the Breast. *JAMA Netw Open*. Published February 24, 2025.
doi:10.1001/jamanetworkopen.2024.61334

Data
Data available: Yes
Data types: Deidentified participant data
How to access data: Clinical, demographic and methylation data were deposited in the NCBI's Gene Expression Omnibus (GEO) database under accession number GSE225845 and is publicly available. (https://www.ncbi.nlm.nih.gov/geo/query/acc.cgi?acc=GSE225845)
When available: beginning date: 10-15-2023

Supporting Documents
Document types: None

Additional Information
Who can access the data: Publicly available.
Types of analyses: Any purpose.
Mechanisms of data availability: Without investigator support.
Any additional restrictions: Clinical, demographic and methylation data were deposited in the NCBI's Gene Expression Omnibus (GEO) database under accession number GSE225845 and is publicly available. The remaining data are available within the article, or as supplementary data, or are available from the authors upon request. Personal identifiers such as neighborhood census tract data and air pollution data cannot be shared.
